# The Interaction Between Pulmonary Fibrosis and COVID-19 and the Application of Related Anti-Fibrotic Drugs

**DOI:** 10.3389/fphar.2021.805535

**Published:** 2022-01-05

**Authors:** Hao Shen, Nu Zhang, Yuqing Liu, Xuerong Yang, Yuanyuan He, Qi Li, Xiaoyan Shen, Yulian Zhu, Yong Yang

**Affiliations:** ^1^ Department of Pharmacy, Sichuan Academy of Medical Sciences and Sichuan Provincial People’s Hospital, School of Medicine, University of Electronic Science and Technology of China, Chengdu, China; ^2^ Personalized Drug Therapy Key Laboratory of Sichuan Province, School of Medicine, University of Electronic Science and Technology of China, Chengdu, China; ^3^ Department of Pharmacy, People’s Hospital of Fushun County, Fushun, China; ^4^ School of Basic Medicine and Clinical Pharmacy, China Pharmaceutical University, Nanjing, China; ^5^ Department of Pharmacy, Ziyang People’s Hospital, Ziyang, China

**Keywords:** pulmonary fibrosis, COVID-19, mechanism, interaction, drug, treatment

## Abstract

COVID-19 is a highly contagious respiratory disease, which mainly affects the lungs. Critically ill patients are easily complicated by cytokine storms, acute respiratory distress syndrome (ARDS), and respiratory failure, which seriously threaten their lives. Pulmonary fibrosis (PF) is a common interstitial lung disease, and its pathogenesis may involve the participation of a variety of immune cells and inflammatory factors. Current studies have shown that patients with COVID-19 may be complicated by pulmonary fibrosis, and patients with pulmonary fibrosis may also be at higher risk of contracting COVID-19 than healthy people. Pulmonary fibrosis is an important risk factor leading to the aggravation of COVID-19 disease. COVID-19 complicated by cytokine storm and ARDS mechanism pathways are similar to the pathogenesis of pulmonary fibrosis. The potential interaction between pulmonary fibrosis and COVID-19 can cause acute exacerbation of the patient’s condition, but the potential mechanism between the two has not been fully elucidated. Most of the drug treatment programs for COVID-19-related pulmonary fibrosis are currently formulated about the relevant guidelines for idiopathic pulmonary fibrosis (IPF), and there is no clear drug treatment program recommendation. This article aims to summarize the relevant mechanism pathways of COVID-19 and pulmonary fibrosis, explore the interrelationships and possible mechanisms, and discuss the value and risks of existing and potential COVID-19-related pulmonary fibrosis treatment drugs, to provide reference for anti-fibrosis treatment for patients.

## Introduction

Severe Acute Respiratory Syndrome Coronavirus type 2 (SARS-COV-2) belongs to the coronavirus subfamily, named coronavirus disease 2019 by the World Health Organization. It is more infectious than Severe Acute respiratory syndrome (SARS) and Middle East Respiratory Syndrome (MERS). Unfortunately, it first broke out in Wuhan, China, in December 2019. Patients infected with SARS-COV-2 may be asymptomatic or present with severe symptoms such as acute respiratory distress syndrome (ARDS) and pulmonary fibrosis. Pulmonary fibrosis (PF) is a common interstitial lung disease, and its pathogenesis may involve the joint participation of various immune cells and inflammatory factors. It is characterized by excessive deposition of extracellular matrix (ECM) in the lung interstitium, which destroys typical parenchymal structure and leads to progressive loss of lung function. Of these, idiopathic pulmonary fibrosis (IPF) is the most common the most severe form, with a median survival of only 2–3 years for untreated patients (Sharif, 2017). The risk factors of idiopathic pulmonary fibrosis and COVID-19 have a lot in common, such as age, sex, smoking history, hypertension, diabetes, obesity, and so on (Li HH. et al., 2021). Both diseases start from lung injury. COVID-19 can lead to more severe ARDS, while IPF usually progresses slowly, and the incidence is hidden.

According to an international multicenter study, the survival rate of hospitalized patients for COVID-19 complicated with idiopathic pulmonary fibrosis or non-idiopathic pulmonary fibrosis was significantly lower than that of patients without pulmonary fibrosis, that is, patients with Interstitial lung diseases (ILD), especially those with fibrotic ILD, faced a higher risk of death after infection with COVID-19 (Drake et al., 2020). A cohort study, which brought into 8,256,161 COVID-19 patients with respiratory diseases, proposed that compared with patients without respiratory diseases, patients with respiratory diseases had a higher risk of hospitalization due to new corona pneumonia (Aveyard et al., 2021). And COVID-19 and IPF have similar risk factors (George et al., 2020). It means that pulmonary fibrosis may be associated with the occurrence of severe COVID-19. COVID-19 Patients with idiopathic pulmonary fibrosis may have a poor prognosis and high mortality.

All ILD, especially IPF, is characterized by acute exacerbation. The pathobiology of acute exacerbation of IPF may be related to inherent defects, an internal acceleration of potential fibrotic conditions, or a response to hidden external events leading to acute lung injury (ALI) and histopathological diffuse alveolar injury (DAD), which make the lungs of IPF patients more vulnerable to external injury than non-IPF. And respiratory failure caused by an acute exacerbation of IPF is related to higher hospital mortality, more than 50% in most cases (Collard et al., 2016). A comparative study in Shanghai (Weng et al., 2019) found that inflammatory cytokines (IL-6, IFN-γ, MIG, IL-17, and IL-9), which play a crucial role in anti-infective responses, were significantly higher in acute exacerbation of IPF than in stable IPF and control groups. And the experimental data showed that the ratio of serum LGM and neutrophils in patients with acute exacerbation of IPF were significantly higher than those with stable IPF. It means that the acute phase of IPF can be triggered by viral infections and greatly enhanced anti-infective immune responses. The COVID-19 Pneumonia and PF are serious diseases characterized by lung injury and repair (Jenkins, 2020). Infection, which is one of the critical causes of ALI, can also cause acute exacerbation of IPF (Weng et al., 2019). Therefore, COVID-19 infection may lead to ALI, accelerate the occurrence of PF, and acute exacerbation of IPF, which may eventually lead to respiratory failure and death. A pre-discharge study of patients with COVID-19 infection suggested that hospitalized COVID-19 patients with basic pulmonary fibrosis diseases may increase their tolerance to low oxygen saturation of the blood (SpO_2_) compared with those without basic pulmonary fibrosis diseases in the absence of subjective dyspnea (Fuglebjerg et al., 2020). It may lead to more unobvious clinical symptoms in COVID-19 patients with PF than those without, and miss the best time to rescue. In general, COVID-19 infection may aggravate PF and increase the risk of death in patients with that.

Therefore, according to various clinical studies, patients with IPF are at high risk of dying from COVID-19, which may lead to the development of pulmonary fibrosis through either virus-induced ALI or cytokine-mediated alveolar damage (Jenkins, 2020). The purpose of this paper is to summarize relevant studies in the existing literature and provide a treatment basis for patients with comorbidity of COVID-19 and IPF.

### The Interaction Mechanism Between COVID-19 and Pulmonary Fibrosis

#### The Route of Infection of SARS-COV-2

Angiotensin converting enzyme 2 (ACE2) is a zinc metalloproteinase, belonging to type I transmembrane protein, whose N-terminal domain-containing carboxypeptidase catalytic site is extracellular and C-terminal tail is intracellular (Donoghue et al., 2000). Spike “S” protein is an exogenous protein expressed in novel Coronavirus and is the key site of virus infection (Huang et al., 2020). When SARS-CoV-2 infects the host cell, its spike “S” protein completes the adsorption process by binding to ACE2 on the host cell, and another cytokine serine protease TMPRSS2 is used to initiate spike “S” protein, both of which eventually lead to the virus invading the target cell (Hoffmann et al., 2020). After entering the cell, the virus unshells, biosynthesizes, assembles, and releases in the cell, eventually causing the host cell to rupture and die. ACE2 gene was widely expressed, including alveolar, trachea, and bronchial epithelial cells, with the highest expression in nasal luminal epithelial cells and decreased expression throughout the lower respiratory tract. Interestingly, this decline parallels a significant decrease in the gradient of SARS-COV-2 infection from upper to lower respiratory tract epithelial cells (Hoffmann et al., 2020; Hou et al., 2020). This creates favorable conditions for the adsorption and invasion of SARS-CoV-2. One of the physiological functions of ACE2 is to convert angiotensin II (Ang II) into angiotensin 1–7 (Ang 1-7), which plays an essential role in controlling cardiovascular and blood pressure (Donoghue et al., 2000). Importantly, Ang II has a fibrotic effect by upregulating the level of pro-fibrosis cytokine transforming growth factor-β1 (TGF-β1), which is involved in transforming fibroblasts into myofibroblasts and extensive collagen deposition (Haulica et al., 2004). However, Ang 1-7 exerts vasodilation, reduces cytokine secretion, inflammation, cardiopulmonary protection, and anti-fibrosis by binding to Mas receptors (Shenoy et al., 2010; He and Garmire, 2020; Sanchis-Gomar et al., 2020). In general, the high affinity of the spike “S” protein on the SARS-CoV-2 envelope to the ACE2 receptor can down-regulate the level of ACE2, increase the level of Ang II and decrease the level of Ang1-7, thus promoting inflammation and pulmonary fibrosis.

Given the difficulty in controlling the high infectivity of SARS-COV-2, we have to suspect that other pathways may infect SARS-COV-2 *in vitro* besides ACE2. CD147, a type II transmembrane protein, is associated with SARS-COV-2 infection (Aguiar et al., 2020; Wang et al., 2020). Wang et al. (2020) showed an interaction between CD147 and the spike “S” protein of SARS-COV-2. They blocked CD147 by CD147 antibody, and the results showed that the amplification of SARS-COV-2 could be inhibited. Thus, they revealed a new viral entry pathway, the CD147-spike protein pathway. Studies have confirmed that, in addition to ACE2 and CD147, SARS-CoV-2 can enter host cells by binding to some other receptors. Recent research hotspots include Cathepsin L1 (Aguiar et al., 2020), integrins αvβ3 and αvβ6 (Calver et al., 2021), and even low-density lipoprotein receptor class A domain containing 3 (LDLRAD3) and C- type lectin domain family 4 member G (CLEC4G) (Zhu et al., 2021). The pathologic consequences of SARS-COV-2 binding to these receptors are uncertain. Still, the effects may greatly contribute to the progression of viral infection and disease, making treatment more difficult.

### The Status Quo of IPF

A survey covering 22 studies in 12 countries estimated the incidence and prevalence of IPF at 0.09–1.30 and 0.33–4.51 per population, respectively (Maher et al., 2021). The occurrence and development of IPF are closely related to aging, genetic background (premature shortening of leukocyte telomere), and epigenetic modification (Fraga et al., 2005; Yang et al., 2019; Duckworth et al., 2021). It is highly correlated with a variety of external factors, including smoking, air pollution, microaspiration, and viral infection, which can lead to repeated damage and repair of alveolar cells (Baccarelli et al., 2009; Launay et al., 2009; Wootton et al., 2011) In the susceptibility stage of pulmonary fibrosis, gene mutations or mutations, epithelial cell replacement lead to telomere shortening, and environmental factors eventually lead to epithelial cell dysfunction (Nunes, 2003). In the initiation stage, molecular mediators of epithelial cell dysfunction such as ER stress; excessive transforming growth factor-β (TGF-β) activation; and growth factor, chemokine, or Wnt secretion lead to EMT, fibrocyte recruitment, and fibroblast differentiation (Nunes, 2003). These molecular mediators will lead to progressive stages in which pathological mesenchymal cells release abnormal types and amounts of stromal proteins that reshape the lung and form scar lung (Nunes, 2003).

### COVID-19 Secondary Pulmonary Fibrosis

#### Multiple Shreds of Evidences of Pulmonary Fibrosis Secondary to COVID-19

A number of studies have directly or indirectly proved that patients infected with SARS-CoV-2 would develop pulmonary fibrosis. CT scans of patients infected with COVID-19 show ground glass shadows that are prone to progression to patchy fibrosis (Ojha et al., 2020). Fibrotic changes in the lungs were found in the autopsy of patients who died of COVID-19 (Liu et al., 2020; Yao et al., 2020). In addition, the factors involved in pulmonary fibrosis such as TGF-β, interleukin-6 (IL-6), and tumor necrosis factor-α (TNF-α) were increased in patients infected with COVID-19. In addition, a meta-analysis of more than 50,000 hospitalized COVID-19 patients showed that the incidence of ARDS was 14.8% (Sun et al., 2020). And more importantly, ARDS patients were very prone to pulmonary fibrosis. Mechanical ventilation, an essential treatment during ARDS, promotes direct penetration of high concentrations of SARS-COV-2 into the lower respiratory tract, potentially causing acute lung injury. Perhaps the underlying mechanisms are epithelial-to-mesenchymal transformation (EMT), changes in cell morphology, and the release of pro-fibrotic mediators such as TGF-β(Gaikwad et al., 2020).

#### Mechanisms of Pulmonary Fibrosis in COVID-19

The abnormal immune system can lead to cytokine storm, resulting in increase plasma concentrations of interleukin 1β, 2, 7, and 10, monocyte chemotactic protein 1, granulocyte-colony stimulating factor, macrophage inflammatory protein 1α, interferon -γ -inducible protein 10 (IP-10), and TNF-α(Hui and Zumla, 2019; Huang et al., 2020; Li et al., 2020). It has been identified that galectin-3 (Gal3) expressed by macrophages, epithelial and alveolar cells in the lung plays a key role in the pro-inflammatory response of microglia (Reyfman et al., 2019). Gal3 binds to and activates TREM2 and TLR4 (Burguillos et al., 2015; Boza-Serrano et al., 2019), both of which have been associated with pulmonary disease, including fibrosis.

Cytokine storm is considered an important factor in accelerating disease progression, leading to severe illness and death. It is one of the main causes of ARDS and multiple organ failure (Chousterman et al., 2017). Due to the abnormal release of matrix metalloproteinases during ARDS, not only the epithelium and endothelium are severely damaged, but also the ECM cross-linking is enhanced, resulting in the change of the structure and composition of interstitial ECM in pulmonary fibrosis (Zemans et al., 2009; Nkyimbeng et al., 2013; Craig et al., 2015; Philp et al., 2018). When alveolar epithelial cells are injured, type II alveolar epithelial cells (ATII cells) proliferate and differentiate into I alveolar cells (ATI cells) to repair the damaged alveoli (Aspal and Zemans, 2020). The evidence supports that the aging and loss of ATII cells are involved in the pathogenesis of IPF(Sisson et al., 2010; Yao et al., 2021). Then, there is reason, to suspect that ATII is also highly associated with fibrosis in virus-infected patients. In addition, alveolar epithelial cells can secrete a series of inflammatory factors, soluble mediators, and remodeling factors, which are closely related to the process of IPF. For example, TGF-β, a factor that stimulates the formation of pulmonary fibrosis, can be activated by the cell membrane integrin of epithelial cells (Jenkins et al., 2006; Tatler and Jenkins, 2012). TGF-β acts on the mesenchymal cells, leading to proliferation and differentiation of fibroblasts into contractile myofibroblasts, and thus causing abnormal production and deposition of extracellular matrix proteins in fibroblasts (Xu et al., 2003; Tatler et al., 2016; Sibinska et al., 2017). Unfortunately, there is new evidence that SARS-COV-2 can bind to cell surface integrins αvβ3 and αvβ6, facilitating their internalization into lung epithelial cells, which in turn accelerates the activity of TGF-β(Calver et al., 2021). This troublesome activation mechanism seems to make the disease harder to control. Except for TGF-β, platelet-derived growth factor (PDGF), connective tissue growth factor (CTGF), interleukin-6 (IL-6), and other cytokines expression increase, also play a certain role in the fibrosis process (Antoniades et al., 1990; Crestani et al., 1994; Pan et al., 2001). Endothelial cells, when injured, transform to a mesenchymal state (EndMT), with an increase in abnormal types and amounts of mesenchymal protein secretion, and at the same time, matrix metalloproteinases degrade the basement membrane collagen that lies beneath the endothelial cells (Gaikwad et al., 2020). These physiological reactions will eventually lead to abnormal accumulation of fibroblasts and myofibroblasts, excessive deposition of matrix proteins in the interstitium of the lung. Then the structure and composition of lung interstitium were changed. Finally, lung tissue sclerosis leads to impaired physiological function and presents a series of clinical symptoms.

#### Risk Factors

When infected with the COVID-19, people may show different symptoms, ranging from asymptomatic to mild such as fever, cough, muscle soreness, or fatigue, and some may become severe like ARDS. Respectively, the proportion of severe and critical cases is as high as 14 and 5% (Wu and McGoogan, 2020). So it is very important to effectively identify the risk factors that promote the development of the disease. Studies have shown that age is an important factor in developing diseases and even death (Zhou et al., 2020; Zhang JJ. et al., 2021). In addition, some studies have shown that male and other chronic diseases, including hypertension, diabetes, obesity, cardiovascular and respiratory diseases, can be positive in the disease leading to exacerbation and death (Zhang J. et al., 2020; Ebinger et al., 2020; Sun et al., 2020). It’s worth noting that pulmonary fibrosis plays an important role in the infection of COVID-19.

#### Predictive Factors of Pulmonary Fibrosis Secondary to COVID-19

There have been many clinical studies on predictors of pulmonary fibrosis secondary to COVID-19. The focus of the study is mainly in two aspects: on the one hand, the level of cytokines including Krebs von den Lungen-6(KL-6), and on the other hand, the CT imaging features.

A growing number of studies support a significant correlation between elevated KL-6 levels and the development of pulmonary fibrosis (Crisan-Dabija et al., 2021; Peng et al., 2021; Xue et al., 2021). For example, a clinical study involving 289 COVID-19 patients finds that KL-6, which is rising earlier than the fibrotic-like change of CT imaging in the lungs, could predict not only the onset of pulmonary fibrosis, but also whether it was reversible (Xue et al., 2021). This is good for both clinicians and COVID-19 patients. However, not all COVID-19 patients have been found a significant association between KL-6 and pulmonary fibrosis. Early in the disease, when extensive lung injury occurs, repair mechanisms of pulmonary epithelial cell is synchronously activated which causing the elevated level of KL-6 (Major et al., 2020). Some studies find that KL-6 were not significantly elevated in some COVID-19 patients with secondary pulmonary fibrosis, possibly because the time when found the occurance of pulmonary fibrosis is later than the time when repair mechanisms was activated and KL-6 was released in large quantities (Xue et al., 2021). In addition, studies have shown that c-reactive protein (CRP), interleukin-6(IL-6), D-dimer and hepatic cytokines (HGF) and C-X-C motif chemokine 13 (CXCL13) are associated with the occurrence of pulmonary fibrosis in patients with COVID-19 (Francone et al., 2020; Yu et al., 2020; Perreau et al., 2021). A study identified two new specific monocyte subsets in patients with severe disease COVID-19:Mono 0 and Mono 5, showing pro-fibrotic and pro-inflammatory characteristics (Zhang Y. et al., 2021). Unfortunately, these studies are all implement in single-center with small sample. Follow-up evaluation of COVID-19 patients from multiple centers is needed to support this conclusion.

Due to inadequate predictive performance of cytokines, radiological assessment may be a method to assess the extent of lung damage caused by COVID-19 and predict the occurrence of pulmonary fibrosis. The features of CT imagine such as interstitial thickening, irregular interfacial, coarse reticular pattern and parenchymal band may be predictive factors of pulmonary fibrosis, and irregular interfacial and parenchymal band can predict the early formation of pulmonary fibrosis (Yu et al., 2020). Multivariable analysis identified age of greater than 50 years, heart rate greater than 100 beats per minute at admission, duration of hospital stay greater than or equal to 17 days, acute respiratory distress syndrome, noninvasive mechanical ventilation, and baseline lung severity score as independent predictors for fibrotic-like changes in the lung at 6 months (Caruso et al., 2021; Han et al., 2021). Similarly, these studies lack rigorous and high-quality evidence. So we can’t make specific recommendations. But based on the above evidence, for patients with advanced age, severe illness, long hospitalization and high CT scores, we should strengthen follow-up after discharge, pay attention to the changes of lung function and carry out health education for them. In the future, more high-quality multi-center large sample data are needed to prove the reliability of these conclusions.

### Whether IPF Patients Are More Susceptible to COVID-19

In recent years, great progress has been made in understanding the pathogenesis of pulmonary fibrosis. Factors like genetics, environmental and aging are involved in the initiation of the fibrosis process. Genome-wide studies have identified many genes associated with the development of IPF, including MUC5B, TERT, FAM13A, DSP, and AKAP13 (John et al., 2021).

Endothelial dysfunction is joint in the IPF, and in patients with pulmonary fibrosis, the level of vascular endothelial growth factor (VEGF) in bronchoalveolar lavage fluid (BALF) is reduced (Koyama et al., 2002). Evidence suggests that the patients with IPF may exhibit susceptibility to SARS-COV-2 due to immune-induced microvascular injury and endothelial cell necrosis (Magro et al., 2006).

Early studies have found that ACE2 is a protective protein that has a positive effect on physiological functions. The regulatory feedback axis composed of ACE2, its product Ang 1-7, and Ang 1-7 receptor plays an important role in pulmonary fibrosis (Li HH. et al., 2021).

Another study (Crisan-Dabija et al., 2020) showed a significant genetic association between IPF patients and elevated expression of AKAP13. This Rho guanine nucleotide exchange factor that regulates the activation of RhoA, which is a molecule that plays an important role in pro-fibrotic signaling pathways. The activation of RhoA drives the fibrotic phenotype, then promotes pulmonary fibrosis. In animal models (pulmonary artery endothelial cells) (Monaghan-Benson et al., 2018), activation of the RhoA/ROCK down-regulates the expression of ACE2, resulting in an imbalance of the renin-angiotensin system (RAS). SARS-COV-2 is known to bind and inhibit ACE2 protein through the affinity and efficiency of its S-spike protein. Given this, IPF patients may be at higher risk.

Studies on pulmonary fibrosis tissue from patients with IPF have shown an increased expression of ACE2 in fibroblasts (Aloufi et al., 2021; Li HH. et al., 2021), and fibroblasts have been identified to carry viral particles of SARS-COV-2 in COVID-19 patients. An *in vitro* study (Li HH. et al., 2021)found that ACE2 and TMPRSS2 were significantly upregulated in the fibroblasts of the IPF and co-expressed in fibroblast-specific protein 1(FSP-1)+ lung fibroblasts. The increased expression of ACE2 in fibroblasts may be related to the feedback regulation of cells. Still, its failure to play a protective role may be related to the activity of receptors, so more studies are needed to explore its specific regulatory mechanism. ACE2, as a protective factor, is widely believed to regulate cell inflammation and apoptosis. The strong affinity between SARS-COV-2 and ACE2 will lead to a decrease in ACE2, while the reduced lung function of IPF patients may increase the possibility of disease aggravation after infection. As for whether the patients with IPF are more sensitive to SARS-COV-2, further clinical studies are needed to find its possible mechanism.

## Potential Agents for Pulmonary Fibrosis Associated With SARS-CoV-2 Infection

### Antiviral Agents

Coronavirus infection could directly promote pulmonary fibrosis. The nucleocapsid protein of Severe Acute Respiratory Syndrome Coronavirus type 1 (SARS-CoV-1) is reported to directly enhance TGF-β signaling, which could strongly promote fibrosis (Zhao et al., 2008). And the nucleocapsid protein of SARS-CoV-2 is over 90% similar to that of SARS-CoV-1 (Tilocca et al., 2020). Therefore, it is critical to reduce the viral load and thus the duration of viral pneumonia in order to prevent fibrosis. Remdesivir is a nucleotide analogue that could selectively block RNA polymerase, thus preventing viral replication. *In vitro* and animal models, remdesivir was found to be effective against SARS-CoV-1 and Middle East respiratory syndrome coronavirus (MERS-CoV). Due to its potential efficacy, the Food and Drug Administration (FDA) has granted remdesivir emergency use authorization for the treatment of COVID-19 (Singh et al., 2020). However, many clinical trials are still underway and its safety must be verified. In addition, the guanine nucleoside analogue ribavirin and the viral assembly inhibitor lopinavir/ritonavir are recommended by *The COVID-19 Diagnostic and Treatment Protocol (Trial Version 8)*, issued by the National Health Commission of the People’s Republic of China. It is reported that lopinavir/ritonavir in combination with interferon-1β or ribavirin might be effective against coronaviruses (Arabi et al., 2020; Dhama et al., 2020). And the recommendation for ribavirin and lopinavir/ritonavir is mainly based on the experience in the treatment of SARS (Chu et al., 2004). The study by Yuan et al. also confirmed that virus-targeted antiviral drugs remdesivir, lopinavir and interferon antiviral drugs IFN-β, especially IFN-β 1b and IFN-β 1a, have strong inhibition effect on viral replication of SARS-COV-2 and significantly reduced viral load. EC50 of remdecivir, lopinavir, IFN-β 1b and IFN-β 1a were 1.04µM, 11.6µM, 31.2 IU/ml and 70.8 IU/ml, respectively (Yuan et al., 2020). Among 4000 COVID-19 patients in the Solidarity trial published by WHO, mortality ratios were 1.16 (95% CI, 0.96–1.39) in the IFN-β 1a plus ritonavir group and 1.12 (95% CI, 0.83–1.51) in the IFN-β 1a alone group (Consortium, 2021). The results showed no effect on mortality reduction of IFN-β 1a. A number of clinical studies have shown that the IFN-β 1b combination has potential value in clinical improvement time, nucleic acid negative conversion time and mortality of COVID-19 patients compared to the control group (using antiviral treatment regimen excluding IFN-β), and no serious adverse reactions have been observed (Hung et al., 2020). However, due to the small sample size, open label randomized controlled trial design and other reasons, it has not yet been able to fully confirm its efficacy and safety, and more high-quality RCT experiments are still needed to evaluate its benefits.

Although SARS-CoV-2 has many similarities with SARS-CoV, the current clinical experience in the treatment of COVID-19 is limited. In addition, the RNA viruses mutate rapidly. The use of antiviral drugs in the early stage of diseases with rapid viral replication is more important than in the middle and late stages with higher levels of inflammatory factors. With the aggravation of the disease, COVID-19 patients are prone to liver and kidney function damage to varying degrees. Currently recommended antiviral drugs also have potential harm to liver and kidney function, which should be paid attention to in clinical use (Wu J. et al., 2020; Pei et al., 2020). Therefore, the use of antiviral drugs has limitations.

### Anti-Fibrosis Drugs

Multiple studies (Noble et al., 2011; Richeldi et al., 2011; Richeldi et al., 2014; Myllarniemi and Kaarteenaho, 2015; Margaritopoulos et al., 2016)have shown that currently available anti-fibrosis drugs nintedanib and pirfenidone can significantly reduce the decline rate of pulmonary function and forced vital capacity (FVC), mitigate the trend of acute exacerbation of fibrosis, and reduced the death rate through inhibiting the production of collagen in activated fibroblasts.

Nintedanib and pirfenidone may alleviate pulmonary fibrosis caused by COVID-19 infection through multiple mechanisms (Umemura et al., 2021). Chronic lung injury caused by overactivity of epidermal growth factor receptor (EGFR) mediated related pathways is one of the main causes of COVID-19 induced fibrosis (Venkataraman and Frieman, 2017). Nintedanib is an EGFR inhibitor that helps prevent excessive fibrotic responses in SARS and COVID-19 (Quartuccio et al., 2020). In addition, studies have found that proteins inhibited by nintedanib are causally related to increased ACE2 expression (Rao et al., 2020). Nintedanib may reduce the expression of ACE2 by inhibiting the expression of related proteins and reducing the body’s response to SARS-CoV-2. Susceptibility. Pirfenidone may inhibit lung damage caused by cytokine storms after SARS-COV-2 infection by significantly reducing serum and lung IL-6 levels (Liu et al., 2017; Zhang C. et al., 2020).

There are still little data on the safety and effectiveness of nintedanib, and pirfenidone in COVID-19 patients with PF, and most clinical trials have yet to be completed. A clinical study designed to observe the efficacy and safety of nintedanib in patients with ARDS induced by COVID found that the application of nintedanib to mechanically ventilated patients with severe new coronary pneumonia can obtain a higher P/F value (according to PaO_2_/FiO_2_ evaluates the severity of the disease) and shorter mechanical ventilation time. In addition, CT volume measurements showed that the percentage of high-density areas in the nintedanib group was significantly lower than that in the control group when patients were not treated with mechanical ventilation. This study suggests that using nintedanib may provide potential benefits for reducing lung injury caused by COVID-19 (Umemura et al., 2021). No clinical data are available on pirfenidone for the treatment of PF in patients with SARS-COV-2 infection.

In addition, as of October 2021, pirfenidone and nintedanib are only marketed in oral form and cannot be used in intubated and mechanically ventilated patients, apparently limiting their use in ICU (George et al., 2020). Moreover, for most patients in the National Health System (NHS), nintedanib or pirfenidone is more expensive to treat. The average adjusted annual costs for patients receiving pirfenidone and nintedanib are 40,000 and 29,000 dollars (Corral et al., 2020).

At present, several clinical trials of pirfenidone inhalation in the treatment of pulmonary fibrosis have been reported. Several studies have shown that pirfenidone has a higher safety as an inhaled delivery agent for the treatment of pulmonary fibrosis. First, inhaled pirfenidone may eliminate the gastrointestinal adverse effects that often occur when administered orally. Second, inhaled powder preparations of pirfenidone have higher photostability and lower risk of phototoxicity compared to oral preparations (Onoue et al., 2013). Third, lower systemic exposure *via* inhaled administration may reduce the risk of hepatic dysfunction due to pirfenidone. A clinical trial evaluating the pharmacokinetics and safety of inhaled pirfenidone solution showed that aerosol pirfenidone was well tolerated in normal volunteers, smokers, and patients with IPF, and no clinically relevant adverse effects related to respiratory rate, spirometry, or oxygenation were observed (Khoo et al., 2020). The main drug-related adverse effect was mild and intermittent cough in a small number of subjects. Furthermore, this study found that systemic pirfenidone exposure at the 100 mg nebulized dose was on average 15 times lower than that reported for the oral licensed dose. And the Capacity 004 phase 3 study of oral pirfenidone demonstrated that adverse events were dose related, not idiosyncratic (Noble et al., 2011).

In addition,a dose/response was demonstrated for the decrease of FVC % predicted and progression-free survival in the CAPACITY 004 trial. And the epithelial lining fluid (ELF) Cmax was reported to be on average 35-fold higher for the 100 mg pirfenidone inhalation dose than the approved oral dose (Khoo et al., 2020). Moreover, a mouse IPF model using bleomycin showed that the efficacy of inhaled pirfenidone was related to peak ELF concentrations rather than AUC(Surber et al., 2014). A recent study using a rat model of paraquat-induced pulmonary fibrosis also showed that the efficacy of inhaled pirfenidone and oral administration was similar, but the inhaled dose was significantly lower than the oral dose (Rasooli et al., 2018). Therefore, the higher local concentration provided by aerosol administration may also lead to the improvement of efficacy. In addition, inhaled pirfenidone offers a new option for patients who are not covenient for oral administration, including patients who are intubated and mechanically ventilated. In summary, further studies of inhaled pirfenidone are necessary to test its long-term safety and efficacy.

At present, there is no clinical trial of inhaled pirfenidone in COVID-19 patients. Although it is mentioned in the literature (George et al., 2020) that “An inhaled formulation of pirfenidone is under evaluation in patients with COVID-19 (NCT04282902)”, in fact, pirfenidone is administered orally in this clinical trial (NCT04282902).

On March 24, 2020, Unco Arendy Therapeutics announced that it is studying OATD-01, a CHIT1 inhibitor, to help treat pulmonary fibrosis in COVID-19 patients. It may have anti-inflammatory activity and may delay the development of pulmonary fibrosis. Studies are currently underway to determine whether patients who have died from COVID-19 have increased CHIT1 expression in their lung tissues, which may lead to a positive effect of OATD-01 on the pulmonary fibrotic development of the disease (Dymek et al., 2018).

### Corticosteroids

Corticosteroids are not recommended for routine use of IPF in several guidelines. Still, systemic Corticosteroids can be used for short-term use in acute exacerbations of IPF to exert anti-inflammatory and immunosuppressive effects of the lung, which can inhibit inflammatory cell infiltration and fibroblast proliferation, reduce alveolar inflammation, and delay the progression of pulmonary fibrosis (Raghu et al., 2015; Homma et al., 2018). As important pathogenesis of acute exacerbation of pulmonary fibrosis, viral infection can easily cause acute lung injury in patients. In addition, patients with the pre-existing progressive pulmonary interstitial disease will have a diffuse pulmonary alveolar injury in severe cases, which is clinically manifested as dyspnea (Collard et al., 2016). This is similar to the lung characteristics seen in critical type COVID-19 patients. Therefore, Corticosteroids can be used to prevent the acute progression of pulmonary fibrosis in PF patients who are prone to severe and critical type COVID-19. The World Health Organization guidelines do not recommend stopping the original systemic Corticosteroids therapy for non-severe COVID-19 patients who have received systemic Corticosteroids therapy. If non-severe COVID-19 patients have clinical symptoms such as increased respiratory rate, respiratory distress, or hypoxemia (similar to symptoms of acute exacerbation of pulmonary fibrosis), systemic corticosteroid therapy is recommended. Systemic Corticosteroids are recommended to treat of severe and critically type COVID-19 patients (Lamontagne et al., 2021).

However, the impact of Corticosteroids on the prognosis of other viral pneumonia still needs clinical attention (Stockman et al., 2006; Arabi et al., 2018). Corticosteroids can delay the clearance of blood and respiratory viruses in SARS/MERS patients (Stockman et al., 2006; Arabi et al., 2018). Corticosteroids do not affect on improving survival, and are accompanied by steroid-related adverse events such as femoral head necrosis, hyperglycemia, and psychosis (Stockman et al., 2006; Arabi et al., 2018). A meta-analysis involving influenza pneumonia patients reported that Corticosteroid use was associated with an increased risk of mortality and secondary infection (Ni et al., 2019). However, Wu et al. said that treatment with methylprednisolone was associated with a reduced risk of death in COVID-19 patients who developed ARDS (Wu C. et al., 2020). Long-term use of low-dose corticosteroids can prevent lung remodeling in ARDS survivors (Gentile et al., 2020). Still, the risk-benefit ratio should be evaluated before use, especially in patients with diabetes, hypertension, and chronic heart failure (Villar et al., 2020). Corticosteroids may delay the clearance of viral RNA and lead to an increased risk of secondary infection (Lee et al., 2004; Arabi et al., 2018), which plays a critical role in the development of fibrosis. Therefore, the use of Corticosteroids should be more cautious. Long-term effects should be emphasized as well as therapeutic products.

### Spironolactone

Spironolactone is an antihypertensive and antiandrogen drug, and it has been reported that it may have important significance in the prevention of pulmonary fibrosis (Zannad et al., 2000; Barut et al., 2016; Yavas et al., 2019). The mechanisms that may have potential benefits for COVID-19 are: 1) increase the circulating level of ACE2 and prevent SARS-COV-2 from entering cells; 2) Block the mineralocorticoid receptors; 3) Down-regulation of TMPRSS2; 4) Anti-inflammatory, antioxidant, anti-fibrosis, and antiviral properties (Cadegiani et al., 2020; Kotfis et al., 2021). ACE2 and TMPRSS2 are key regulators of SARS-COV-2 cell entry. It has been reported that the elevation of the physiological corticosteroid receptor activator aldosterone is associated with fibrosis and a high inflammatory response (Stone et al., 2020; Salama et al., 2021). Therefore, spironolactone has potential value in preventing or reducing pulmonary fibrosis after COVID-19. In addition, Atalay et al. confirmed the effectiveness of spironolactone in treating ALI in rats (Atalay et al., 2010). And Lieber et al. found that spironolactone can reduce acute pneumonia caused by bleomycin or lipopolysaccharide (Lieber et al., 2013). Still, related experiments were performed on animal models such as rats or other rodents, and its conclusions have apparent limitations. In addition, there is currently a lack of direct clinical research results showing that mineralocorticoid receptor antagonists are beneficial for pulmonary fibrosis after viral infection. In the future, further trials are needed to evaluate the potential benefits of spironolactone to COVID-19 and to clarify its mechanism further.

### Cytokine Inhibitors

#### Tocilizumab

Tocilizumab is a humanized monoclonal antibody against IL-6, which can bind to the soluble IL-6 receptor on the cell membrane and inhibit the function of IL-6 (Gautret et al., 2020). IL-6 is not only the main therapeutic target for the treatment of COVID-19 complicated by cytokine storm syndrome (Wan et al., 2020), it is also a pro-fibrotic factor produced in the Th2-type immune response. Studies have observed that fibroblasts induce fibroblasts to transform into myofibroblasts by secreting IL-6, which promotes the occurrence and development of pulmonary fibrosis (Kobayashi et al., 2015). At present, whether tocilizumab can be used in the treatment of new crowns is still controversial. The multicenter trial results showed that tocilizumab reduced the risk of COVID-19 hospitalized patients progressing to mechanical ventilation or death on the 28th day (Salama et al., 2021). A randomized trial in Brazil stopped early due to a significant increase in deaths within 15 days in the tocilizumab treatment group compared with the placebo group (Veiga et al., 2021). Another North American study showed that tocilizumab did not prevent intubation or death of early COVID-19 hospitalized patients. However, there is currently a lack of clinical studies evaluating the long-term prognostic effect of tocilizumab in avoiding or reducing COVID-19-related pulmonary fibrosis.

#### TGF-β1 Inhibitors

##### Histone Deacetylase Inhibitors

HDAC inhibitors have been reported to show good anti-fibrotic effects mainly through suppressing TGF-β1 signaling (Atalay et al., 2010). Fibroblasts to myofibroblasts differentiation generally mediated by TGF-β1 require HDAC4 (Glenisson et al., 2007). Several studies (Glenisson et al., 2007; Barter et al., 2010; Korfei et al., 2015) have shown that HDAC could epigenetically regulate TGF-β-mediated gene expression, which leads to pulmonary fibrosis. Therefore, it is possible to treat pulmonary fibrosis with HDAC inhibitors. HDAC inhibitor tubastatin successfully ameliorated pulmonary fibrosis in murine bleomycin-induced pulmonary fibrosis model, particularly triggered by TGF-β(Saito et al., 2017). In addition, a study has demonstrated that panobinostat is superior to pirfenidone against IPF-derived fibroblasts (Korfei et al., 2018).

Murthy et al.(Murthy et al., 2021) said that COVID-19 recovered patients who show unresolved patchy areas of opacification, interstitial thickening and early signs of fibrosis during the follow-up chest CT after discharge should be considered for HDAC inhibitors treatment to reduce the possibility of the development of pulmonary fibrosis. Early treatment with HDAC inhibitors for the secondary/late consequences of SARS-CoV-2 infection will help reduce complications/mortality and improve the quality of life of patients for COVID-19 recovered patients.

### CD147 Inhibitors

SARS-CoV-2 invades host cells through the CD147 receptor, which is present in multiple cell types of the lung and is highly expressed in type II alveolar cells and macrophages at the edge of the fibrotic zone. A phase 2 clinical trial to prevent COVID-19 by blocking CD147 is underway in China (Ulrich and Pillat, 2020), which is testing a humanized form of the CD147-specific antibody meplazumab. Furthermore, transient transfection of normal human lung fibroblasts to overexpress CD147 could significantly increase TGF-β1-induced cell proliferation and the expression of α-smooth muscle actin, a marker of myofibroblasts (Guillot et al., 2006), thus anti-CD147 antibodies could inhibit TGF-β1-induced proliferation and differentiation of fibroblasts into myofibroblasts that are induced by TGF-β1. Therefore, blocking CD147 is potentially valuable for the prevention of the pulmonary fibrosis due to COVID-19.

#### Poly-(ADP-Ribose) Polymerase Inhibitor

SARS-CoV-2 infection can induce PARP activation in lung tissue of asthmatic patients; different preclinical animal models (Ghonim et al., 2015a; Carlile et al., 2016) showed that PARPI attenuated pulmonary fibrosis caused by SARS-CoV-2 pulmonary inflammation. Its possible mechanism of improving COVID-19 pulmonary fibrosis is to protect cells from death by preventing cytokine storms (excessive activation of macrophages) (Curtin et al., 2020). Several animal model studies (Ghonim et al., 2015b) (Sethi et al., 2019; Sahu et al., 2020) have shown that PARPI, including Olaparib, can reduce the expression of IL-6 and IL-1β in many organs like the lung. Therefore, PARPI can be applied as a potential treatment for pulmonary fibrosis caused by SARS-CoV-2 infection.

### Propolis

SARS-CoV-2 invasion to host cells can be prevented or reduced by Propolis extract and its components reducing TMPRSS2 expression and ACE2 anchoring (Kaur et al., 2020). Furthermore, propolis block kinase PAK-1, which increases during lung inflammation and fibrosis (Berretta et al., 2020), and PAK1 inhibitors were reported to rescue the immune system and help resist virus infection (Maruta and He, 2020). Propolis, comprising its components, can help prevent immunosuppression in the early stage of the disease and reduce the host’s excessive inflammatory response by blocking excessive IL-6, IL-2, and JAK signals in the later stage (Nile et al., 2020).

### Galectin-3 Inhibitor

One of the main stages of COVID-19 is the excessive inflammation stage, during which immune cells release Gal3 (Boza-Serrano et al., 2019). And it has been reported that the level of Gal3 in proliferative T cells of COVID-19 patients is elevated (Liao et al., 2020). Gal3 is a carbohydrate-binding protein expressed by macrophages, epithelial cells, and alveolar cells in the lung (Reyfman et al., 2019). Gal3 binds and activates TLR4 and TREM2, a process associated with lung diseases and pulmonary fibrosis (Boza-Serrano et al., 2019). TREM2 is expressed by macrophages and is associated with fibrosis (Liao et al., 2020). TLR, which is essential for the antiviral response, can lead to solid inflammation associated with the expression of interferon-related genes, interleukins, chemokines, and Gal3 (Guo et al., 2020). The Gal3 inhibitor TD139 provided by Galecto Biotech has proven safe and effective for patients with IPF (NCT03832946). The clinical trials using TD139 for the treatment of COVID-19 patients are still under study (NCT04473053).

### Chinese Medicine Treatment Strategy for Pulmonary Fibrosis in Convalescent Sequelae of COVID-19

Current studies have proved that traditional Chinese medicine prescription is effective in the treatment of pulmonary fibrosis, and the combination of Chinese and Western medicine is obviously superior to western medicine alone therapy in delaying the decline of pulmonary function and improving the quality of life (Gan et al., 2020). In the theory of traditional Chinese medicine, COVID-19 is caused by damp toxin and epidemic gas, and the disease location is mainly in the lung as well as the spleen and stomach. Active intervention of traditional Chinese medicine in the early stage of recovery can promote the absorption of pulmonary lesions and improve pulmonary function.

Based on the studies on traditional Chinese medicine treatment of IPF and SARS-associated pulmonary fibrosis, there are many traditional Chinese medicine prescriptions that can significantly relieve the symptoms of pulmonary fibrosis and improve lung function. Huaxian Decoction is a classic prescription for the treatment of pulmonary fibrosis, which is anti-inflammatory effect may be mediated by inhibition of inflammatory factor TNF-α、TGF-β, and inhibit NF-κB activation of inflammatory signaling pathways, thus effectively alleviate inflammatory reaction, reduce clinical symptoms, improve lung function, and reduce the levels of serum laminin, hyaluronic acid and other pulmonary fibrosis indicators (Chen et al., 2016; Gan et al., 2020). Yiqi Huoxue Guben decoction can delay the decline of lung function, improve respiratory symptoms, improve activity endurance and improve the quality of life (Gan et al., 2020). Huqihuoxue Decoction can inhibit the expression of TGF-β and its protein, reduce the lung collagen deposition in patients with idiopathic pulmonary fibrosis, thus relieve the symptoms of patients. Peiyuan Quyu decoction can improve the clinical symptoms and lung function index of phlegm-stasis IPF, alleviate the progression of acute exacerbation of the disease, and significantly improve exercise endurance (Changhui, 2019). Qingjin Yifei decoction can reduce the expression of VEGF mRNA and PDGF, thereby reducing capillary permeability, dilating pulmonary artery veins, and improving vascular remodeling.

After the treatment of COVID-19 in the acute stage, the clinical outcomes of TCM dialectics mainly divided into three categories: For those with residual pathogens accompanied by qi and Yin deficiency syndrome, should be treated to nourish qi and Yin, and supplemented with the prescription of invigorating qi and promoting blood circulation, which is suitable for shengmai Decoction, Shashen Maimendong Decoction and Wuye Lugen Decoction; For those with deficiency of lung and spleen, the method is to replenish qi and strengthen the spleen, aromatize and remove dampness, and add or subtract Liujunzi Decoction; For those with qi deficiency, blood stasis and obstruction of lung collaterals, supplementing qi, activating blood circulation and collaterals are the main treatment methods, which can be combined with Buyang Huanwu Decoction and Xuanpu Hua Decoction (Gan et al., 2020).

## Adjustment of the Drug Treatment Regimen for PF Patients Infected With COVID-19

The current drug treatment methods for PF, especially IPF, mainly include anti-fibrosis therapy, antacid therapy (proton pump inhibitors, H_2_ receptor antagonists), hormone therapy, immunomodulatory therapy. When patients with pulmonary fibrosis are infected with SARS-COV-2, the original anti-fibrosis treatment plan should be adjusted according to the antiviral treatment plan and pathophysiological state of the patient. The suggestions and precautions of drug adjustment for anti-fibrosis therapy, anti-acid therapy, and immunomodulatory therapy are discussed.

### Anti-Fibrosis Therapy

National Institute for Health and Clinical Excellence (NICE) and British Thoracic Society (BTS) guidelines recommend that patients receiving anti-fibrosis therapy should not stop using pirfenidone and nintedanib. There is currently no evidence that the use of pirfenidone and nintedanib increases the risk of SARS-CoV-2 infection or COVID-19 aggravation.

The following points should be noted in the application of nintedanib and pirfenidone. The two drugs often have gastrointestinal side effects that are similar to the symptoms of COVID-19 (diarrhea, fatigue, loss of appetite) and should be carefully differentiated in clinical use. Secondly, these two drugs have certain hepatotoxicity. COVID-19 patients, especially in severe cases, often report liver damage (George et al., 2020). The liver function of COVID-19 should be closely monitored during use, and anti-fibrosis drugs should be stopped in time if patients develop acute liver function damage. Thirdly, studies have shown that patients with COVID-19 have an increased risk of venous vascular embolism, especially pulmonary embolism. At the same time, viral infection leads to the acute progression of original pulmonary fibrosis. Meanwhile, viral infection leads to the sharp progression of pulmonary fibrosis, and the body is prone to hypercoagulability due to an imbalance between coagulation and fibrinolysis (Abou-Ismail et al., 2021). At this time, if the anticoagulant warfarin is used, attention should be paid to discontinue the use of nintedanib for anti-fibrosis therapy. It is a vascular endothelial growth factor receptor (VEGFR) inhibitor. The simultaneous use of anticoagulants will increase the risk of bleeding in patients. Finally, pirfenidone is strictly prohibited for patients with severely impaired renal function. If the patient’s glomerular filtration rate (eGFR) is less than 30 ml/min/1.73 m^2^, pirfenidone should be discontinued (George et al., 2020).

### Antacid Therapy

Gastroesophageal reflux is a risk factor of pulmonary inflammation caused by aspiration or slight aspiration of acidic or non-acidic reflux substances into the lungs, and it is also an important mechanism of pulmonary fibrosis caused or aggravated by sustained lung injury (Trachalaki et al., 2021). Gastroesophageal reflux can be observed in 90% of patients with idiopathic fibrosis, regardless of the presence of gastrointestinal symptoms (Tobin et al., 1998). The ATS/ERS/JRS/ALAT clinical practice guidelines are weak recommendations for the use of conventional antacid therapy (PPI or H_2_ receptor antagonists) in patients with pulmonary fibrosis, suggesting that conventional antacid therapy may improve pulmonary function and survival in patients with pulmonary fibrosis, and may reduce the cost of treatment for the progression of pulmonary fibrosis (Raghu et al., 2015). A clinical study analyzed the impact of throat reflux disease (a subtype of gastroesophageal reflux disease) on COVID-19 hospitalized patients. Among the 95 hospitalized patients, 37 patients had throat reflux disease (reflux symptom index, RSI >13). The median RSI score of severe and critical COVID-19 patients was significantly higher than that of ordinary patients, which suggesting that throat reflux disease may be a risk factor for severe or critical COVID-19 patients (Jiang et al., 2020). Anti-acid therapy is indeed indicated for COVID-19 patients. However, the results of a meta-analysis showed that conventional PPI treatment of COVID-19 is related to the prolonged hospital stay of patients, and it is more likely to lead to severe consequences such as admission to the intensive care unit, mechanical ventilation, acute respiratory distress syndrome or death (Li GF. et al., 2021). Recent studies have shown that famotidine can improve the clinical symptoms of non-hospitalized COVID-19 patients (Janowitz et al., 2020), and is associated with lower mortality and lower risk of death from endotracheal intubation in hospitalized patients (Mather et al., 2020). Its mechanism may be related to H_2_ receptor-mediated immunomodulatory effect on mast cell histamine-cytokine crosstalk (Mather et al., 2020). Therefore, FOR COVID-19 patients, especially those in critical condition, PPI should be used cautiously, and famotidine can be considered the first choice.

### Immunomodulatory Therapy

Convalescent plasma, COVID-19 human immunoglobulin, and tocilizumab are recommended for COVID-19 immunotherapy acc according to the Chinese Diagnosis and Treatment Protocol for Novel Coronavirus Pneumonia (Trial Edition 8). Tocilizumab can treat patients with extensive bilateral lung disease or severe patients with elevated IL-6 levels. However, the guidelines issued by the National Institutes of Health (NIH) indicate that there is insufficient research evidence for IL-6 inhibitors for the treatment of COVID-19. One study suggested that the TGF-β pathway in the pathogenesis of IPF could be mediated by IL-6 (Epstein Shochet et al., 2020). Tocilizumab does suggest that it can be used in patients with pulmonary fibrosis complicated with COVID-19, which has potential value in inhibiting the progression of pulmonary fibrosis and preventing cytokine storms arising from immune imbalance through immunomodulatory effects. Milo Gatti et al. conducted a pharmacovigilance evaluation on tocilizumab. The results of the study found that it easily leads to liver damage in patients. In severe cases, acute liver failure, fulminant hepatitis, and liver necrosis can occur. Severe and critically COVID-19 patients have varying degrees of liver and kidney damage. The benefit to risk ratio should be fully considered, and the continued use of tocilizumab should be carefully considered. Mild patients can continue to use it while monitoring liver function (Gatti et al., 2021).

## Discussion

At present, with the COVID-19 outbreak in full swing, the number of infected people is growing and challenging to control. Any progress in scientific research requires the accumulation of time. Even though scientific researchers around the world are dedicated to studying the novel coronavirus infection, the progress and outcome of COVID-19, and related treatment drugs, less than 2 years after the outbreak, there are still many research gaps and many mechanisms. The problem is difficult to explain clearly. Most importantly, drugs for the treatment of COVID-19 are still being explored and discussed, and we have not found the specific drugs we hoped. At present, the treatment of COVID-19 associated pulmonary fibrosis can only be based on the existing IPF treatment regimen. The study by Umemura et al. evaluated the efficacy and safety of nintedanib for pulmonary fibrosis induced by COVID-19 in ICU patients with severe COVID-19. The results showed that nintedanib combined with favipiravir can significantly reduce the mechanical ventilation time of ICU patients with COVID-19, the percentages of high-attenuation areas at liberation from mechanical ventilation. There was no statistically significant difference in 28 days mortality from the control group (Umemura et al., 2021). In terms of safety, there were no statistically significant differences in severe, moderate and mild liver failure and gastrointestinal reactions in nintedanib combined with favipiravir compared with favipiravir alone, but the incidence of mild and moderate acute liver failure was slightly higher. Although statistical tests showed no statistical difference between the two groups, the sample size of the experiment was small (n = 60), which might lead to sampling error. Meanwhile, the subjects in this study were only ICU patients, and the benefits and risks of nintedanib in patients with mild disease cannot be evaluated for the time being. In addition to the clinical study mentioned above, few clinical trials of drug therapy for COVID-19-related pulmonary fibrosis have published its results (Kotfis et al., 2021). Therefore, it is currently impossible to accurately evaluate the efficacy and safety of anti-fibrosis drugs in the treatment of COVID-19-related pulmonary fibrosis. In the process of clinical treatment, medical staff should be more cautious, strictly control the indications for drug use, carefully select the anti-fibrosis drug treatment plan, and give priority to the safety of patients after drug use. Based on the existing clinical evidence, it is more recommended to choose the first-line anti-fibrosis drugs pirfenidone and nintedanib, which are recommended by authoritative guidelines and have sufficient evidence in medicine. Currently, there is no clear interaction between the COVID-19 antiviral drugs recommended by the mainstream internationally and nintedanib and pirfenidone. In addition, nintedanib and pirfenidone are not immunosuppressive agents themselves, and there is currently no evidence that they will prolong the clearance time of SARS-CoV-2 *in vivo* (George et al., 2020).

There are various limitations of existing drugs in clinical practice, which should be paid attention to in the process of clinical use. First of all, there is an overlap between the adverse reactions of anti-fibrosis drugs and those related to COVID-19 treatment, and the incidence of adverse reactions in patients is easy to increase when combined. For example, although nintedanib and pirfenidone are both authoritative drugs recommended to treat idiopathic pulmonary fibrosis, there is currently a lack of clinical studies on their treatment of SARS-CoV-2 infected pulmonary fibrosis. Here we emphasize the safety of the drug. The adverse reactions of nintedanib and pirfenidone (including gastrointestinal reactions and liver toxicity, etc.) coincide with the clinical symptoms of COVID-19. In addition, remdesivir, lopinavir, ritonavir, and other clinically recommended antiviral drugs are also prone to gastrointestinal reactions and liver function damage in patients (Cao et al., 2020; Spinner et al., 2020; Mahajan et al., 2021). This seems to be very detrimental to the patient. Secondly, there are contradictions between anti-fibrosis drugs and new coronary treatment-related treatments. For example, nintedanib, as a VEGFR inhibitor, is likely to increase the risk of bleeding, which seems to contradict with the increased risk of pulmonary embolism in patients with COVID-19. Therefore, in the clinical use of nintedanib and anticoagulants, the patient’s condition should be thoroughly evaluated. The pros and cons should be weighed to avoid the occurrence of acute disease progression and serious adverse events. Finally, there are some similarities between the anti-fibrosis treatment plan and the COVID-19 treatment plan. Patients with COVID-19 associated pulmonary fibrosis seem to have more pointers to use drugs recommended for both diseases (such as glucocorticoids, acid-suppressing drugs). Still, the potential risks of drugs should also be paid attention. For example, current clinical evidence favors glucocorticoids to control the acute progression and prevent severe and critical illness. A meta-analysis that included patients with influenza pneumonia reported that glucocorticoid use was associated with an increased risk of mortality and secondary infection (Ni et al., 2019). Severe and critically ill COVID-19 patients are more likely to be mechanically ventilated and prone to ventilator-associated pneumonia. Glucocorticoids should be used under close observation in such patients. At the same time, glucocorticoids can delay the clearance of SARS/MERS patients from blood and respiratory viruses due to immunosuppressive effects (Stockman et al., 2006; Arabi et al., 2018). Therefore, it should be noted that larger doses of glucocorticoids may delay the clearance of SARS-CoV-2 and prolong the course of patients.

We look forward to better clinical trials of targeted drugs targeting fibrosis pathways. We look forward to researchers around the world developing new drugs with both effectiveness and safety in response to the continuing outbreak of COVID-19.
